# Intermittent Fever, Progressive Weight Gain, and Personality Changes in a Five-Year-Old Girl: Unusual Paraneoplastic Syndrome due to Presacral Ganglioneuroma

**DOI:** 10.1155/2016/2743576

**Published:** 2016-06-20

**Authors:** Chao Yang, Chang-chun Li, Jun Zhang, Xiang-ru Kong, Zhenzhen Zhao, Xiao-bin Deng, Liang Peng, Shan Wang

**Affiliations:** Department of Pediatric Surgical Oncology, Children's Hospital of Chongqing Medical University, 136 Zhongshan 2nd Road, Yuzhong District, Chongqing 400014, China

## Abstract

Ganglioneuromas are rare tumors in the neuroblastoma group. Paraneoplastic syndrome (PNS) due to presacral ganglioneuromas was hardly reported in previous literature. Here, we reported that a case of a 5-year-old girl with a presacral ganglioneuroma presented with PNS, who presented with intermittent fever, progressive weight gain, and personality changes. Our report revealed intermittent fever, progressive weight gain, and personality changes may represent rare paraneoplastic syndromes in ganglioneuromas.

## 1. Introduction

Ganglioneuromas are rare tumors in the neuroblastoma group [1]. They are benign lesions arising from sympathetic ganglion cells and complete surgical excision is considered to be curative [2–4]. Presacral ganglioneuromas are rare and so far less than 20 cases have been reported in the literature [5]. Paraneoplastic syndrome (PNS) due to presacral ganglioneuromas was hardly reported in previous literature. Here, we report a case of a 5-year-old girl with a presacral ganglioneuroma presented with PNS.

## 2. Case Report

A 5-year-old girl sought medical advice in September 2012 because of a history of progressive weight gain (30 Kg, BMI 29.4), short stature (0.96 m; −2 SDs), obesity, moon face, buffalo hump for more than 1 year, personality changes for 6 months, and intermittent fever for 1 month. She displayed increased weight since she was 4 years old. There was neither hypertension nor headache history observed. Personality changes were noticed 6 months ago and she presented with soliloquy and was annoyed and irritable. Parorexia was also observed. She presented with intermittent fever one month ago with the highest temperature of 40°C, accompanied with convulsions three times and recurrent cough. She was admitted to her local hospital, the diagnosis of pneumonia and obesity was confirmed, and antibiotic and mannitol were administrated; however, the symptoms were not relieved and she was transferred to our hospital for further treatment.

**Figure 1:**
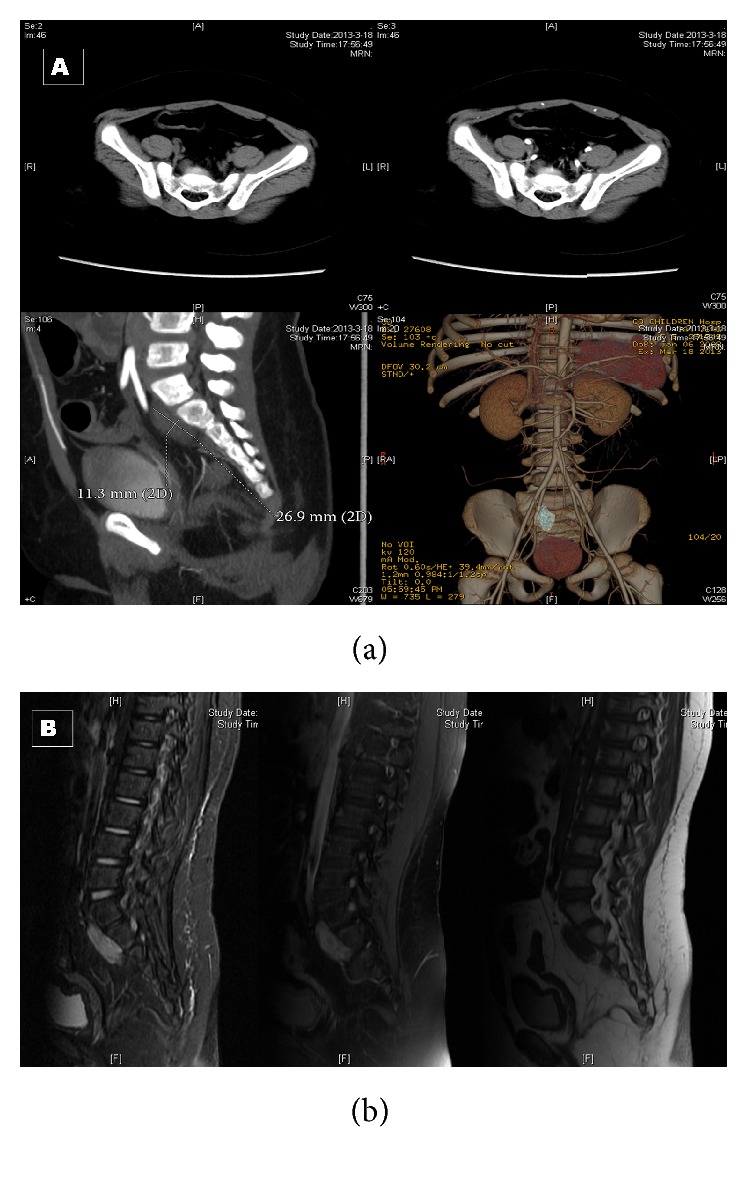
(a) The CT scan abdomen showed a dishomogeneous mass (11.2 mm × 26.9 mm × 11.3 mm) with obvious enhancement located before the sacral vertebra. (b) MR images revealing a presacral ganglioneuroma originated from sacral canal in S1-2.

**Figure 2:**
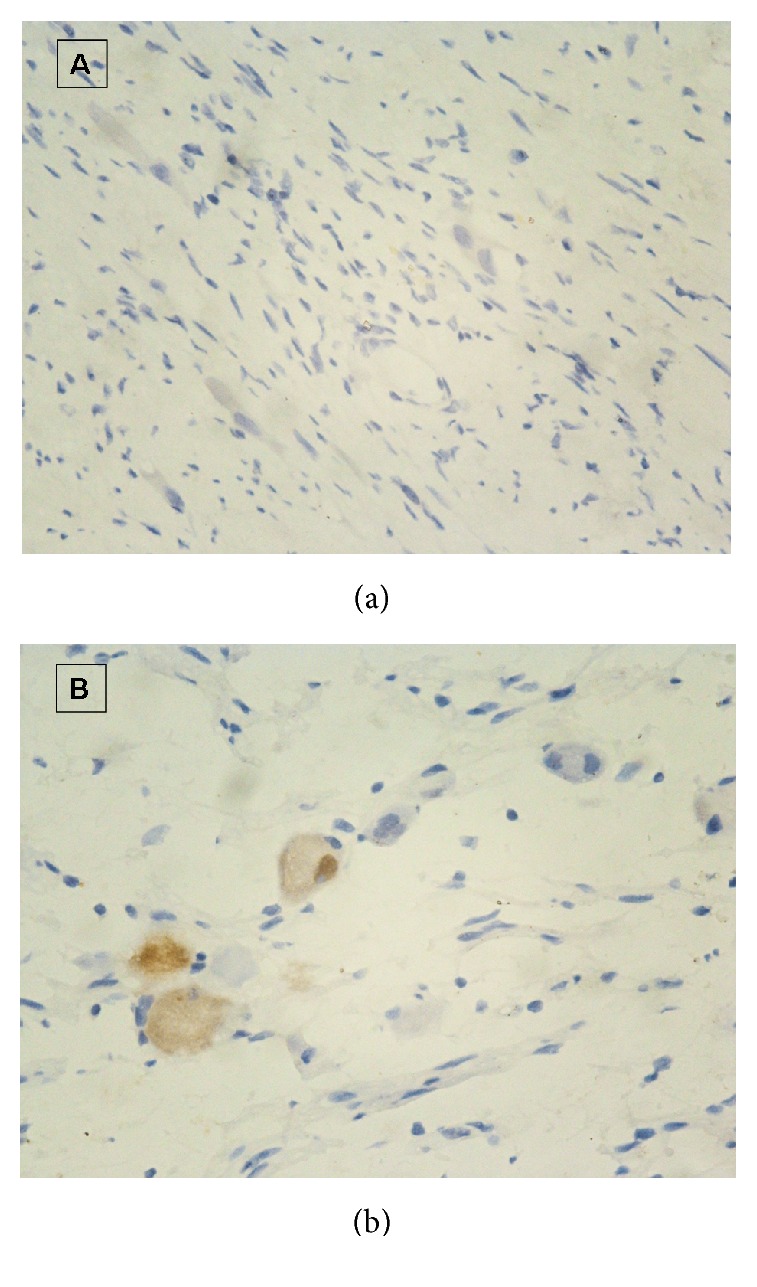
(a) Photomicrograph of a ganglioneuroma showing large mature neurons in a neuromatous proliferation including spindle-shaped Schwann nuclei. (b) Positive immunostaining of neuroganglional cells with anti-ACTH antibodies.

After administration, physical examination and neurologic examination were negative. The blood pressure was 133 over 86. Routine blood tests were made and tumor markers were detected (CEA, CA 19-9, CA 125, AFP, and HCG), but all values were normal. Serum cortisol was 2852 nmol/L, 2201 nmol/L, and 1250 nmol/L, 8/16/24 h, respectively (normal, 138–690 nmol/L). Plasma adrenocorticotropic hormone (ACTH) levels were 162 pg/mL, 108 pg/mL, and 89 pg/mL, 8/16/24 h, respectively (normal, <46 pg/mL). Vanilmandelic acid (VMA) was within the normal range. Karyotype was normal. Pituitary thyroid magnetic resonance imaging (MRI) was negative, and abdomen-thorax computed tomography (CT) scan with contrast enhancement scan revealed a dishomogeneous mass (11.2 mm × 26.9 mm × 11.3 mm) with obvious enhancement located before the sacral vertebra. Pelvic MRI confirmed the origin of the lesion from sacral canal in S1-2 (Figure 1) and excluded any sign of sacral or coccygeal metameric infiltration or osteolysis. The diagnosis of Castleman disease was suspected, and PNS due to this mass was also considered. In case of adrenal crisis postoperatively, hydrocortisone was administrated 3 days before and after surgery. The patient was submitted to surgical laparotomy: hypogastrium transverse incision and transperitoneal exposure were performed; small bowel, distal sigmoid, and rectum and their mesenteries were retracted to expose the tumor lesion. A mass measuring 3 cm × 2 cm × 2 cm was revealed and tenaciously stuck to sacral plane. Intraoperative frozen section excluded the malignancy of the lesion. A complete resection was performed. Histopathologic examination confirmed the diagnosis of ganglioneuroma, consisting of a majority of Schwann cells with variable amounts of collagen and some groups of ganglion cells and no neuroblast. Immunostaining for ACTH was focally positive (Figure 2).

After surgery, the patient's serum cortisol returned to the normal range, with values of 380 nmol/L, 320 nmol/L, and 260 nmol/L, 8/16/24 h, respectively, and blood pressure also returned to the normal range, and she had a regular postoperative hospital stay without complications. The temperature returned to normal 4 days after surgery and she was discharged on the 10th postoperative day. After a 6 mo follow-up, her personality changes were improved gradually, and no sign of progressive weight gain was observed. Follow-up of at least 5 years was planned to detect early recurrence if any.

## 3. Discussion

Histologically ganglioneuromas are considered to be part of the neuroblastoma group together with neuroblastomas and ganglioneuroblastomas [6, 7]; the cell of origin is derived from embryonic neural crest cells, which are destined to form autonomic nerve tissue. Although neuroblastomas are composed of neuroblasts, ganglioneuromas consist of mature ganglion cells and other mature tissues and are considered benign. It is believed that these tumors represent a continuum and that ganglioneuromas are the final stage in the maturation of neuroblastoma cells [6–9].

Arising along the sympathetic chain, ganglioneuromas are commonly localized in the posterior mediastinum followed by retroperitoneum, cervical region, and adrenal gland [7]. The presacral location is very rare. Ganglioneuromas have usually a mean diameter of 7 cm, so our patient is a rare case for both its presacral location and size [1].

These tumors may show hormonal activity. Hypertension, flushing, diarrhea, and virilization may occur as a result of the secretion of catecholamines, vasoactive intestinal polypeptide, or androgenic hormone [10]. Occasional reports of ACTH/CRH-producing ganglioneuromas presenting clinically as Cushing's syndrome were reported.

PNS in children is rare; mature neuroblastomas, that is, ganglioneuroblastomas, are known to produce peptides that may cause PNS, which are mainly neurological, such as cerebellar encephalopathy, opsoclonus-myoclonus, constipation, diarrhoea, and encephalomyelitis/sensory neuronopathy [11]. However, there was little report about PNS due to ganglioneuromas. To our knowledge, ganglioneuromas have been reported to secrete vasointestinal peptide and also can present as Cushing's syndrome [12, 13]. Except ectopic Cushing syndrome, our patient presented a complicated symptom of intermittent fever, progressive weight gain, and personality changes, which was barely observed in ganglioneuroma patients. The body temperature dropped to normal after surgery, which indicated the fever might be due to PNS such as an interleukin secretion by the tumor. After surgery, the serum cortisol and blood pressure returned to the normal range, and her personality changes were improved gradually; also no sign of progressive weight gain was observed at follow-up, which suggested an evidence of these symptoms that were caused by this tumor.

Imaging investigations had not hinted that the etiological diagnosis of these symptoms was a ganglioneuroma. On CT scanning, ganglioneuromas show no or mild heterogeneity, low attenuation and may exhibit calcification [14]. The patient's CT scan showed a dishomogeneous mass with obvious enhancement, which did not support the diagnosis of ganglioneuroma. Combined with her complicated clinical manifestations, Castleman disease was suspected. However, pathological examination confirmed the diagnosis of ganglioneuroma, which suggested ganglioneuroma should be considered in this situation.

## 4. Conclusion

Presacral ganglioneuromas are rare benign lesions in pediatrics and may present with various symptoms. Surgery is the primary means of diagnosis and treatment. Ganglioneuroma should be considered as a differential diagnosis in patient with presacral tumor accompanied with various clinical manifestations and Cushing syndrome.

## Abbreviations

PNS: Paraneoplastic syndrome
VMA: Vanilmandelic acid
CT: Computed tomography
MRI: Magnetic resonance imaging.
